# Mammary epithelial cell phenotype disruption *in vitro *and *in vivo* through ERalpha36 overexpression

**DOI:** 10.1371/journal.pone.0173931

**Published:** 2017-03-16

**Authors:** Charlène Thiebaut, Clémence Chamard-Jovenin, Amand Chesnel, Chloé Morel, El-Hadi Djermoune, Taha Boukhobza, Hélène Dumond

**Affiliations:** CNRS-Université de Lorraine, UMR 7039, Centre de Recherche en Automatique de Nancy, BP70239, Vandœuvre-lès-Nancy, France; University of South Alabama Mitchell Cancer Institute, UNITED STATES

## Abstract

Estrogen receptor alpha 36 (ERα36) is a variant of the canonical estrogen receptor alpha (ERα66), widely expressed in hormone sensitive cancer cells and whose high expression level correlates with a poor survival prognosis for breast cancer patients. While ERα36 activity have been related to breast cancer progression or acquired resistance to treatment, expression level and location of ERα36 are poorly documented in the normal mammary gland. Therefore, we explored the consequences of a ERα36 overexpression *in vitro* in MCF-10A normal mammary epithelial cells and *in vivo* in a unique model of MMTV-ERα36 transgenic mouse strain wherein ERα36 mRNA was specifically expressed in the mammary gland. By a combination of bioinformatics and computational analyses of microarray data, we identified hierarchical gene networks, downstream of ERα36 and modulated by the JAK2/STAT3 signaling pathway. Concomitantly, ERα36 overexpression lowered proliferation rate but enhanced migration potential and resistance to staurosporin-induced apoptosis of the MCF-10A cell line. In vivo, ERα36 expression led to duct epithelium thinning and disruption in adult but not in prepubescent mouse mammary gland. These phenotypes correlated with a loss of E-cadherin expression. Here, we show that an enhanced expression of ERα36 is sufficient, by itself, to disrupt normal breast epithelial phenotype in vivo and in vitro through a dominant-positive effect on nongenomic estrogen signaling pathways. These results also suggest that, in the presence of adult endogenous steroid levels, ERα36 overexpression *in vivo* contributes to alter mammary gland architecture which may support pre-neoplastic lesion and augment breast cancer risk.

## Introduction

Twenty years ago, steroid hormones, especially sex hormones, were shown to stimulate gene transcription through binding a transcription factor in the cell nucleus called a nuclear steroid receptor. The so-called “genomic estrogen signaling” is mediated by direct actions of nuclear-localized estrogen receptors (ERs: ERalpha and ERbeta) as ligand-induced transcription factors [1,2]. However, estrogen activities are also mediated through nongenomic signaling which involves extranuclear events such as activation of various protein kinases mediated by membrane associated ERs or the G protein-coupled estrogen receptor (GPER) [3].

In 2005, Wang and colleagues [4] identified and cloned a 36-kDa variant of ERα, ERα36, which is mainly located on the plasma membrane and mediates nongenomic estrogenic signaling. ERα36 differs from the canonical 66-kDa estrogen receptor alpha (ERα66) by the lack of both AF-1 and AF-2 transcription activation domains, and a truncated ligand-binding domain deleted from helix9 to helix12. These structural characteristics are consistent with the fact that ERα36 has no intrinsic transcriptional activity and suggest that it may have a spectrum of ligand selectivity different from the ERα66 one. Although the ability of ERα36 to interact directly with any ligand is still debated, tamoxifen has been demonstrated to bind ERα36 as an agonist like 17β-estradiol, which triggers proliferation, migration and apoptosis escape of breast cancer cells [5–8]. Moreover, ERα36 is generated from a promoter located in the first intron of the ESR1 gene, indicating that its expression can be regulated independently from ERα66 and consistent with the findings that ERα36 is expressed in ER-negative breast cancer cells that lack ERα66 expression [4,9]. Even in the absence of known agonists, a high expression of ERα36 is a marker of poor survival prognosis for breast cancer patients [10, 11].

Consistent with its subcellular localization, ERα36 can mediate membrane-initiated signaling through physical interaction with the EGFR/Src/Shc complex, or functional coupling with GPER or HER2, depending of the tumor cell tested [12–15]. In various types of cancerous cell lines (endometrial, ER positive or negative breast cancer cells, seminoma cells…), ERα36 may activate calcium release, the PKC, MAPK/ERK or the PI3K/AKT signaling pathways responsible for cell proliferation, migration and survival [16–18].

ERα36 mRNAs have been found in normal ovary, uterus, breast and testis tissues as well as endothelial and vascular smooth muscle cells, kidney, cartilage, bone, lung and heart [18]. A sexual dimorphism of protein location was reported in the osteoarticular system where ERα36 appears to associate with plasma membrane only in females. However, the ERα36 physiological role(s), ligand(s) and associated transactivation mechanism(s) in those tissues remain to be determined [19, 20]. Despite ERα36 activity have been related to breast cancer progression and acquired resistance to tamoxifen and chemotherapy breast tumor treatment, precise expression level and location of ERα36 is still missing in the normal mammary gland [21].

The mammary gland is an epidermal appendage, originating from ectodermal and mesodermal elements, whose development is a step by step sequential process which begins in utero at 10.5 day post-coïtum (dpc) in mouse and 4 to 6 weeks in human and ends at adulthood with lactation and involution [22, 23]. A rudimentary mammary tree is present in newborn females, whereas regression occurred under androgen production during male fetal development [24]. During female puberty, the epithelium forms into a branching, bilayered ductal structure, consisting of an outer basal myoepithelial layer of cells and an inner luminal cell layer [24]. Epithelial estrogen and progesterone were shown to be responsible for ductal elongation and side branching, respectively [25, 26]. Several studies based on KO mouse phenotype analyses demonstrated that the estrogen receptors ERα and ERβ are dispensable before puberty but required for a correct mammary gland development and function in adulthood [26, 27]. Indeed, ERα is expressed in epithelial and stromal cells and mediate duct elongation whereas ERβ is mostly involved in cell differentiation and function during gestation and lactation [27, 28]. Despite an abundant and detailed literature dealing with ERα36 expression and function in breast cancer, no data has been reported to date about a putative role of ERα36 in mammary gland normal development or the molecular and cellular consequences of its transactivation.

In this work, we explored the consequences of a potential ERα36 overexpression in normal mammary epithelial cells in vitro and in a unique model of transgenic mouse strain, in which ERα36 is expressed specifically in the mammary gland. We identified proliferation, survival and migration as the main functions modulated by ERα36 overexpression through STAT3 pathway, and assumed that this could lead to predispose normal mammary epithelial cells to neoplastic like transformation and augment breast cancer risk.

## Material and methods

### Animal husbandry

Mice were housed under constant conditions at a temperature of 22±1°C, a humidity of approximately 40% and a 10h-light/14h-dark cycle. Food (SAFE, France) and water disposed in glass bottles were supplied *ad libitum*. All experimental procedures including the study of a total of 120 animals were approved by the French Minister of Research Committee for animal experiment (APAFIS#2168–2015110518268051 v5).

### ERα36 transgenic mouse strain

ERα36 transgenic (Tg) founders were obtained from the Mouse Clinical Institute, Illkirsch-Graffenstaden, France by injecting an MMTV/ERα36 chimeric construction into one of the pronuclei of a hybrid B6SJLF2 background (S1A Fig). The transgene is a transcriptional fusion composed of the MMTV promoter sequence from pGL4.36 plasmid (Promega) and the complete ERα36 human cDNA sequence [4] as previously described for EGFR in Brandt [29]. The transgene copy number was determined by droplet digital PCR using specific primers, and a clone bearing a single copy of the MMTV-ERα36 transgene was retained, in order to avoid transgene silencing and obtain 50% hemizygote transgenic offspring when crossed with wild-type (wt) C57BL/6J strain.

The presence or absence of the ERα36 transgene was assessed at weaning (postnatal day 21, PND 21) by means of genomic DNA extraction from ear pinna samples and further real time PCR determination (forward 5’-GCTTCGATGATGGGCTTACT-3’; reverse 5’- CTAAACTGGGAGGTACTAGTCC-3’). A sequence from chromosome 17 was used as a reference (forward 5’-AAGGAGCAAGGTGGCTTACA-3’; rev 5’-TGAGAAGGGTACCGTCACGG-3’).

### ERα36 transgene expression in transgenic strain

The male transmission of the transgene was preferred rather than the female one in order to avoid a maternal effect of ERα36 expression, through potential physiological or behavioral alterations. Seven week old hemizygote ERα36 transgenic males from F1 to F3 generations (6 males per generation) were crossed with seven week old C57BL/6J wt virgin females obtained from Charles River Laboratories, France. The mammary gland specific ERalpha36 mRNA expression was determined in F1 to F4 litters. Mammary gland, uterus, ovary, testis, heart, liver, brain, kidney and salivary gland were then dissected from 21 day or 4 month old F2 to F4 progenies (at least 5 unrelated animals for each age and generation), RNA extracted and checked for ERalpha36 expression by qRT-PCR (Forward 5’-ATGAATCTGCAGGGAGAGGA; reverse 5’-GGCTTTAGACACGAGGAAACC-3’). The RPLPO gene was used as a reference (forward 5’-GGCGACCGTGAAGTCCAACT-3’; reverse 5’-CCATCAGCACCACAGCCTTC-3’). In wt mice, we detected no ERα36 expression, whatever the organ, the sex or the age of the animal tested. In transgenic mice, none of the tested organs except the mammary gland did express the transgene (not shown). The protein expression in adult mammary gland (4 month old females) was confirmed by western blot analysis (S1B Fig). The sex ratio and transmission rate of the transgene were determined by means of male/female and transgenic littermate number determination in each litter and generation in a total of 171 animals (S1C Fig).

### Mammary gland whole mounts

To prepare the whole mounts, the mammary glands were dissected from animals anesthetized with a lethal dose of pentobarbital, spread onto glass slides and stained as described by Vandenberg et al. [30]. A dedicated Matlab program, adapted from Tylcz et al. [31] was used to quantify mammary tree extension and branching. First, the ducts in all the images are enhanced using the multiscale filtering method of Frangi et al. [32] (S2A Fig). The objectives are (i) to increase the difference in pixel intensities between ducts and background tissues, and (ii) to prevent structures breaks. The ducts are then detected by segmentation (hard thresholding) and skeletonization. Finally, the mammary network is quantified in terms of tree extension, branching and amount of sprouts in F2 and F4 animals (F2wt: N = 4, F2 Tg: N = 6, F4wt: N = 6, F4Tg N = 5). (S2B Fig).

### Histology

To prepare paraffin sections, mammary glands were fixed with Davidson fixative [33] for 24h at room temperature, dehydrated and embedded with paraffin (VWR). Seven micrometer sections were cut on a Leitz rotary microtome (Leica) and mounted on Superfrost slides (Fisher Scientific); the entire mammary gland was sectioned at once. Sections were stained with hematoxylin/eosin/methyl green to determine the presence of epithelial cords. Sections with visible cords were used for further analysis. In order to determine mammary ducts parameters, only orthogonally sectioned ones were chosen. By using NIS-elements BR4.20.00 imaging software (Nikon), epithelium and stromal thickness as well as lumen width were measured along both the larger and smaller diameters of the ducts. At less 5 duct sections from 5 separate slices were measured in each mammary gland. A total of 27 animals were used for histology (at weaning wt: N = 6; Tg N = 8; adults wt: N = 6; Tg N = 7).

### Cell culture

MCF-10A cells were purchased in 2015 from ATCC^®^ (CRL-10317) and maintained in DMEM/F12 (GIBCO) supplemented with 5% horse serum (HS), 1% Glutamine 1% Penicillin/Streptomycin and a mix of EGF (epidermal growth factor), Cholera toxin, hydrocortisone and insulin as described by Soule et al. [34].

### Transient transfection and stable cell line establishment

Stable MCF-10A cell lines transfected by pCDNA3.1-ERα36 (pCDNA3.1vector containing the complete cDNA sequence of ERα36) or the empty expression vector were obtained as previously described [17]. Corresponding cell lines were named MCF-10A/ERα36 and MCF-10A/Zeo, respectively. The transfected cell lines were then subcloned and checked for ERα36 expression by RT-PCR (S3A Fig), western blot (S3B Fig) or immunofluorescence analysis before each experiment (S3C Fig).

### Microarray experiment

Transcriptional profile analyses of MCF-10A/ERα36 and MCF-10A/Zeo cells cultured for 2 days in standard medium were performed in triplicates on Affymetrix GeneChip U133 2.0 by the GenomEast Platform (IGBMC, Strasbourg, France). Biotinylated cRNA targets were prepared using the Ambion “MessageAmp™ Premier RNA Amplification Kit" according to the Instruction Manual P/N 4386269 Revision D (Revision Date: May 16, 2008), starting from 200 ng of total RNA extracted with RNeasy Mini kit (Quiagen). Following fragmentation, 10 μg of cRNAs were hybridized for 16 hours at 45^°^C, 60 rpm on Human GeneChip® HG-U133 plus 2.0 arrays (Affymetrix). The chips were washed and stained in the GeneChip® Fluidics Station 450 (Affymetrix) using the FS450_0004 script and scanned with the GeneChip® Scanner 3000 7G (Affymetrix) at a resolution of 1.56 μm. Raw data (.CEL Intensity files) were extracted from the scanned images using the Affymetrix GeneChip® Command Console (AGCC) version 4.0. CEL files were further processed with Affymetrix Expression Console software version 1.3.1 to calculate probeset signal intensities, using Robust Multi-array Average (RMA) algorithms with default settings.

### Real-time PCR analysis

RT and real-time PCR analyses were performed as previously described [17]. The following primers were used for qRT-PCR: *RPLPO* forward (Fw) 5’-GGCGACCGTGAAGTCCAACT-3’, *RPLPO* reverse (Rev) 5’-CCATCAGCACCACAGCCTTC-3’, *CDH1* forward (Fw) 5’- TGCCCAGAAAATGAAAAAGG -3’, *CDH1* reverse (Rev) 5’- GTGTATGTGGCAATGCGTTC -3’, *CDH2* forward (Fw) 5’-ACAGTGGCCACCTACAAAGG-3’, *CDH2* reverse (Rev) 5’-CCGAGATGGGGTTGATAATG-3’. Assays were performed at least in triplicate, and the mean values were used to calculate expression levels, using the ΔΔC(t) method referring to RPLPO housekeeping gene expression.

### Crystal violet assay

The crystal violet assay was performed in 24-well plates seeded with 5x10^2^ cells per well. After each well was washed with PBS, the cells attached to the bottom of the plate were fixed and stained with 0.4% crystal violet solution in 2% ethanol for 30 min. After the plate was washed with water and dried, crystal violet was solubilized in 10% acetic acid and the absorbance at 570 nm was measured by a microplate reader (Victor x3, Perkin-Elmer).

### Western immunoblotting

Western blots were performed as described previously [17]. The following primary antibodies were used: anti-Cyclin D1 (#2922, Cell Signaling), anti-ERα36 (CY1109, Cell Applications), anti-PARP 1 cleaved (552596, BD Pharmingen) and anti-Caspase 7 cleaved (#9494, Cell Signaling), anti- Caspase 3 cleaved (#9664, Cell Signaling). The anti-β Actin antibody (sc1615, Santa Cruz Biotechnology), anti-Glyceraldehyde-3-Phosphate-DesHydrogenase (GTX100118, Genetex) or anti-α-tubulin (GTX102079, Genetex) were used as a control. Protein expression profiles were revealed with Clarity Western ECL Substrate (Biorad) and banding quantification was performed using the Quantity One Chemidoc XRS software (Biorad).

### Immunofluorescence

Immunofluorescence was performed as described previously [17]. The following primary antibodies were used: anti-cytochrome c **(**sc13561, Santa Cruz Biotechnology), anti-NFκB p65 (GTX102090, GeneTex), anti-β-catenin (E247, Epitomics #1247-s), anti-E-cadherin (GTX100443, GeneTex), anti-N-cadherin (TA326835, OriGene), anti-PTEN (#9188, Cell Signaling), anti-phospho-ERK1/2 (#4370, Cell Signaling) and anti-STAT3 (#12640, Cell Signaling). Goat anti-rabbit secondary antibody was coupled to AlexaFluor 555 (Invitrogen). Images were obtained with DS-Ri1 Nikon camera and Eclipse80i Nikon microscope and quantifications were performed using NIS-Elements BR 4.20.00 software (Nikon).

### Scratch-wound assay

Scratch assays were performed using the Ibidi-culture inserts (Ibidi®/Biovalley) following the manufacturer instructions. Cultures were then washed to remove detached cells and debris. Quantification of wound mean width were performed at t = 0 and t = 6h of culture by phase-contrast image analysis with NIS-elements BR 4.20.00 software (Nikon).

### TUNEL assay

TUNEL assay was performed using the Apo-BrdU-IHC in situ DNA fragmentation Assay kit (BioVision, USA) following the manufacturer instructions for the staining of cell preparations fixed on slides adapted for the use of AlexaFluor 555 (Invitrogen) goat anti-mouse secondary antibody. Nuclear DNA was stained with Hoechst (bisBenzimide H33342 Trihydrochloride, Sigma-Aldrich). Quantification of stained cell number was performed with NIS-elements BR 4.20.00 software (Nikon).

### Annexin V apoptosis detection assay

Cell apoptosis was assayed by using the BD Annexin V: FITC Apoptosis Detection system. Propidium iodide and FITC fluorescence of the stained cells were automatically acquired and analyzed with a BD FACSCalibur^TM^ (BD BioSciences).

### Statistical analysis

All data are summarized as the mean ± SEM and results considered statistically significant *P*<0.05. Statistical analyses were performed with Matlab vR2014b software (MathWorks) by using Student t-test for unpaired samples with a significant p-value threshold below 5%.

## Results

### Microarray and bioinformatic analysis of differentially expressed genes in MCF-10A/ERα36 *versus* MCF-10A/Zeo cells

In order to determine which cell function could be altered by ERα36 overexpression, we analyzed and compared the transcriptional profiles of MCF-10A/ERα36 and MCF-10A/Zeo cells. 8022 transcripts were significantly up- or down-regulated (absolute variation factor ≥ 2.3 in triplicate RNA samples, corrected p-value *P*<0.05 with Benjamini-Hochberg method) between the 2 cell lines. Corresponding genes were termed “differentially expressed genes” (DEGs). The overall strategy of DEG list analysis is described in S4A Fig.

### Gene Ontology enrichment analysis of differentially expressed genes

The online tool MSigDBv5.0 (Molecular Signature Database http://www.broadinstitute.org/gsea/msigdb/) [35, 36] was used to achieve Gene Ontology (GO) enrichment analysis. The overlap between DEG list (see above) and GO gene sets derived from the Biological Process part of GO was computed. The four functions mostly affected by ERα36 overexpression were signal transduction (GO:0007165; *P* = 1x10^-44^), cell proliferation (GO:0008283; *P* = 1x10^-9^), cell surface receptor linked signal transduction (GO:0007166; *P* = 1x10^-14^) and apoptotic process (GO:0006915; *P* = 1x10^-9^). Proportion of genes allocated to one GO gene set affected by the overexpression are 43.3%, 43.3%, 41.8% and 43.2%, respectively.

### Pathway enrichment of DEGs

Pathway enrichment analysis was also achieved with the online tool of the KEGG database (http://www.genome.jp/kegg/). The four best overlaps between the DEG list and KEGG database signaling pathways were identified for the “PI3K-AKT Signaling pathway” (hsa04151), “MAPK Signaling pathway” (hsa04010), “cAMP Signaling pathway” (hsa04024) and “Jak/STAT Signaling pathway” (hsa04630) (*P* -value<10^−3^). Proportion of genes allocated to one KEGG signaling pathway affected by the overexpression are 36.6%, 39.8%, 38.4% and 41.1%, respectively.

### Identification of key DEG regulators downstream of ERα36

Thereafter, Ingenuity Pathway Analysis (IPA^®^) and Matlab^®^ software were used to identify intermediate factors that may contribute to the target gene expression changes observed after ERα36 overexpression.

The DEG list was submitted to the IPA^®^ software which predicted ESR1 gene (encoding ERα36) and 17β-estradiol (ERα36 agonist) as key upstream regulators of the DEG list. The direct and indirect functional links (affected, activated and inhibited) between the DEGs and the predicted upstream regulators are described by the IPA^®^ Software as 3 adjacency matrices in a digraph collecting the gene interactions.

In order to identify intermediate regulators acting downstream ERα36 and involved in the control of each function previously identified by MSigDB^®^ analysis, gene-regulator networks, corresponding to each of the four specific functions (see GO gene sets above), were built using the Matlab^®^ software. By running a specific Matlab program on adjacency matrices, we identified among the upstream regulators determined via IPA (i) the first level of intermediate regulators that directly regulate DEGs, and (ii) the second level of intermediate regulators that control directly the intermediates from the first level and thus indirectly the DEGs. Iterative identification of intermediate regulator levels was performed until no more element could be added into the regulator set.

Then, a digraph was drawn for each function representing its regulator hierarchical network for the considered DEGs. In the networks, the DEGs, intermediate or upstream regulators are represented as nodes. The vertices indicate the regulation relationship between the nodes. An example of such a network is described in S4B Fig.

The common intermediate regulators (i.e. nodes) involved in the four functions were determined by comparing the four networks. For each node of any network, we associated an integer which indicated the number of networks in which it can be found. This led to the identification of 24 common intermediate factors that could mediate ERα36 dependent target gene regulation. Among them, we selected for further experimental validation, the intermediates involved into the signaling pathways identified from KEGG database.

**Fig 1:**
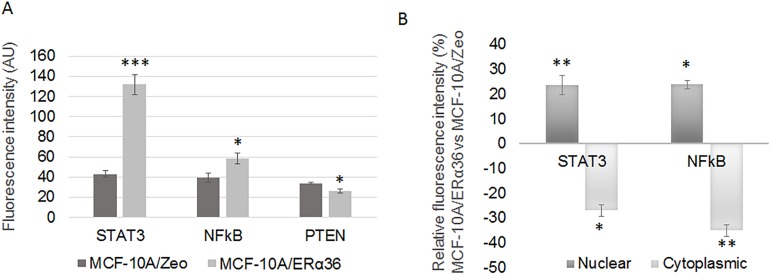
ERα36 overexpression modulates MAPK/ERK1/2, NFkB and JAK2/STAT3 signaling pathways in MCF-10A cells. Expression levels of PTEN, Phospho-ERK1/2 (P-Erk), NFκB and STAT3 were measured by immunofluorescence with specific antibodies: anti-PTEN, anti-Phospho-ERK1/2, anti-NFκB p65, anti-STAT3 in MCF-10A/Zeo and MCF-10A/ERα36 cells. Total, nuclear and cytoplasmic average fluorescent signal intensities (AlexaFluor 555) are quantified from 5 cells in at least 5 separate fields per condition. Each bar represents mean ± S.E.M. N = 3 independent experiments. *: *P*<0.05, ***: *P* <0.001. A. Average fluorescence of PTEN, Phospho-ERK1/2, NFκB and STAT3 in whole MCF-10A/Zeo and MCF-10A/ERα36 cells. B. Ratios of nuclear or cytoplasmic average fluorescence of STAT3 and NFkB intensities in MCF-10A/ERα36 versus MCF-10A/Zeo cells indicating nuclear relocalization following ERα36 overexpression.

The expression and the localization of key effectors, associated with PI3K/AKT (PTEN, PI3K), MAPK/ERK1/2 (phosphorylated ERK1/2), JAK/STAT (STAT3) or NFkB (p65) signaling pathways, were studied *in vitro* by immunofluorescence. Fluorescence intensity quantification indicated that each protein was expressed in MCF-10A/Zeo cells at a similar level. Nevertheless, ERα36 overexpression significantly induced STAT3 (206%) and NFkB (49%) expression but decreased PTEN (23%) expression (Fig 1A confirmed by western blot in S5A Fig). A nuclear translocation of NFkB (24%) and STAT3 (23%) was also observed in MCF-10A/ERα36 cells compared to MCF-10A/Zeo cells (Fig 1B, S5B Fig). This was consistent with an activation of the NFkB and JAK2/STAT3 signaling pathways.

In parallel, the accuracy of the *in silico* derived predictions was also verified by examining the MCF-10A/ERα36 versus MCF-10A/Zeo cells phenotypes. First, we focused on proliferation and apoptosis that are 2 cellular functions previously highlighted by the GO enrichment analysis and then we studied the migration/invasion that could be affected by the TGFB1 intermediate factor.

### ERα36 expression and cell division

**Fig 2:**
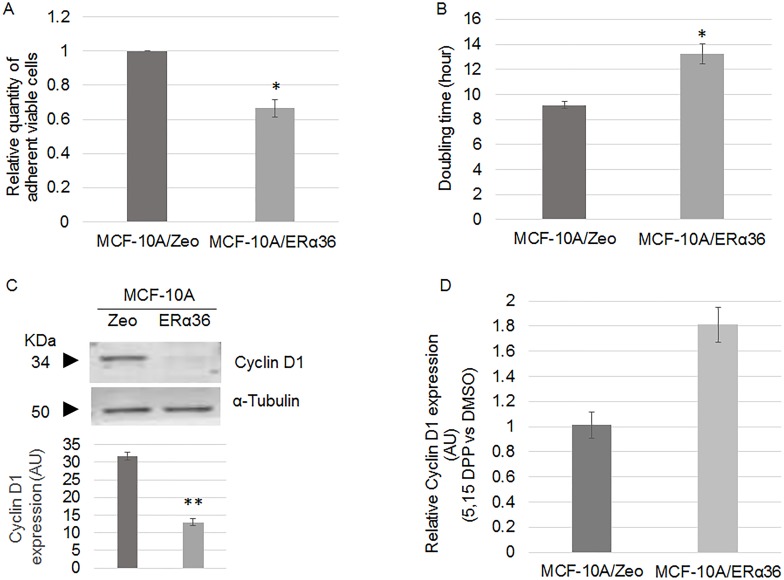
ERα36 overexpression lowers MCF-10A cell proliferation rate. A. Quantification of MCF-10A/Zeo and MCF-10A/ERα36 cell viability by crystal violet assay after 48h culture. ERα36 overexpression triggers a 35% decrease of cell proliferation. Each bar represents mean ± S.E.M. N = 3. *: *P*<0.05. B. Doubling time of MCF-10A/Zeo and MCF-10A/ERα36 cells was measured by crystal violet cell counting at t = 0 (immediately after seeding), t = 24h, 48h and 72h of culture. Given the measurements of living cells at t = 72h and t = 0, doubling time was calculated assuming a constant growth rate. Each bar represents mean ± S.E.M. N = 4. *: *P*<0.05. C. Representative western blot analysis of Cyclin D1 expression in MCF-10A/Zeo and MCF-10A/ERα36 cells (left panel). α-Tubulin was used as a loading control. Quantification of corresponding band intensity (right panel) indicate a 60% decrease of cyclin D1 expression in MCF-10A/ERα36 compared to MCF-10A/Zeo cells. Each bar represents mean ± S.E.M. N = 3. **: *P*<0.01. D. Relative Cyclin D1 expression in MCF-10A/Zeo and MCF-10A/ERα36 cells after 24h DMSO or 10μM 5,15 DPP exposure. Results depicted in the histogram are represented as 5,15 DPP versus DMSO ratio. In the presence of 5,15 DPP, cyclin D1 protein expression increases by 81% only in MCF-10A/ERα36 cells.

Quantification of viable adherent cells by crystal violet staining indicated a 35% decrease of cell number in MCF-10A/ERα36 compared to MCF-10A/Zeo cells (Fig 2A). Consistently, MCF-10A/ERα36 doubling time was 40% higher than MCF-10A/Zeo one (Fig 2B), probably due to a 31% longer S phase, as measured by flow cytometry (not shown). Expression of cyclin D1, a key marker of cell cycle entry was also decreased by 60% in MCF-10A/ERα36 compared to MCF-10A/Zeo cells (Fig 2C). Since this could appear to contradict to the previous observation that STAT3 mediates ERα36 signaling, we measured cell proliferation and cyclin D1 expression after a 24 h treatment with 5,15 DPP, an inhibitor of STAT3 activity. 5, 15 DPP exposure had no effect on both parameters in the control MCF-10A cell line but triggered a 40% increase of cell number and a 81% increase of cyclin D1 expression in MCF-10A/ ERα36 cells. These results suggest that ERα36 overexpression may slower cell division in a STAT3 dependent manner.

### ERα36 expression and apoptosis escape

**Fig 3:**
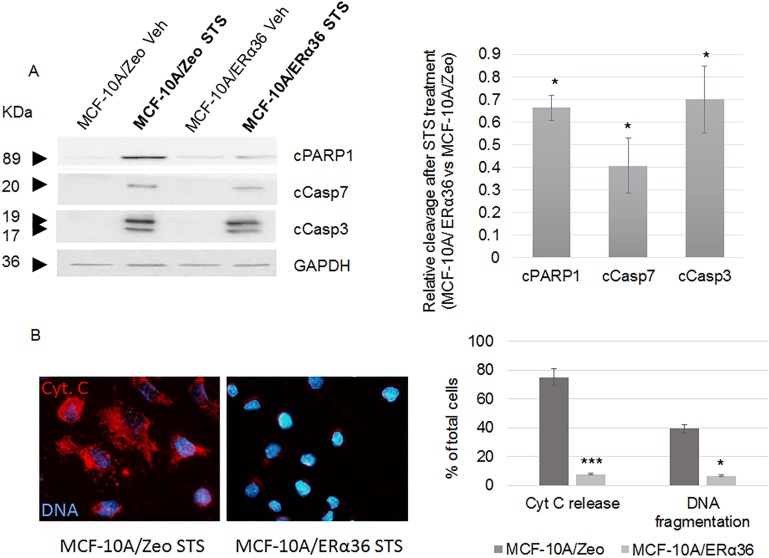
ERα36 overexpression stimulates apoptosis resistance. MCF-10A/Zeo and MCF-10A/ERα36 cells were exposed to 0.25μM staurosporin (STS) or vehicle (Veh) for 6 hours. A. Cleavage of PARP1 (cPARP1), Caspase 7 (cCasp7) and Caspase 3 (cCasp3) were evaluated with specific antibodies (left panel). GAPDH was used as a loading control. Results depicted in the corresponding histogram are represented as STS versus Vehicle ratio (right panel). ERα36 overexpression triggered a significant 34%, 60% and 30% decrease of PARP1, Caspase 7 and Caspase 3 cleavage, respectively. Each bar represents mean ± S.E.M. N = 4. *: *P* <0.05. B. Cytochrome c (Cyt. C) release (red, AlexaFluor 555) and DNA fragmentation were respectively assessed by immunofluorescence and TUNEL assay after STS exposure in MCF-10A/Zeo and MCF-10A/ERα36 cells (left panel), then quantified as shown in the corresponding histogram (right panel). No cytochrome c release or DNA fragmentation can be detected in untreated cells (not shown). Each bar represents mean ± S.E.M. N = 3. *: *P* <0.05, ***: *P* <0.001.

MCF-10A/Zeo and MCF-10A/ERα36 cells were exposed for 6 h to DMSO (vehicle) or staurosporin (STS), a potent inducer of apoptosis. Fig 3A indicates that DMSO treated cells displayed no apoptosis. STS treatment triggered a significant expression of apoptotic markers in both cell lines with a respective 34%, 60% and 30% reduction of PARP 1, caspase 7 and caspase 3 cleavage in MCF-10A/ERα36 compared to MCF-10A/Zeo cells. In order to confirm this result, we performed an FITC-Annexin V assay after both cell line STS treatment. After a 6-hour exposure, we detected less than 5% of stained cells in either control or ERα36 overexpressing MCF-10A cells. An extended exposure time up to 24h led to a 10.85% reduction of apoptotic cell number in MCF-10A/ERα36 compared to MCF-10A/Zeo cell line. No staining was detected in DMSO exposed control cells. We also performed a TUNEL assay after 24h vehicle or STS exposure: less than 0.3% vehicle treated cells were stained whereas 39.3% and 6.5% fragmented nuclei were detected in STS exposed MCF-10A/Zeo or MCF-10A/ERα36, respectively (Fig 3B). Finally, a representative immunofluorescence image and the corresponding quantification of Fig 3B indicate that cytochrome c release by 75% in ERα36 overexpressing MCF-10A cells. Taken together, these data strongly suggest that ERα36 overexpression leads to a higher resistance to STS-induced apoptosis.

### ERα36 expression and migration potential

**Fig 4:**
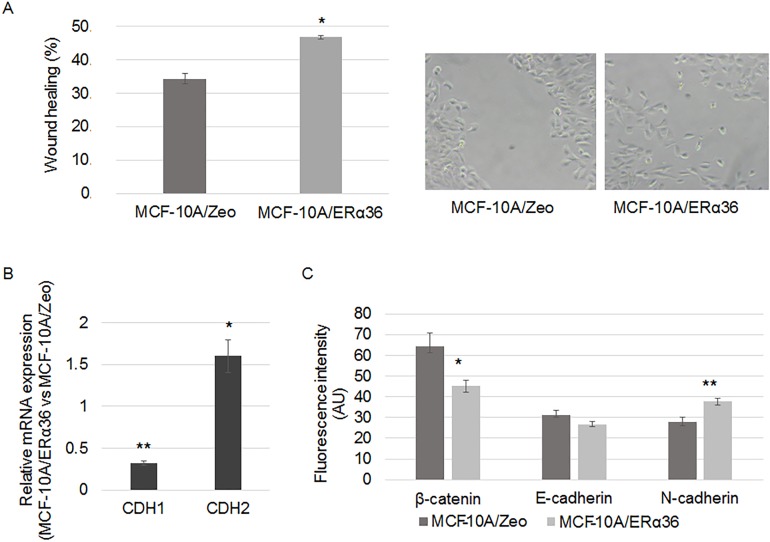
ERα36 overexpression enhances migratory potential. A. A wound was performed on a confluent monolayer culture of MCF-10A/Zeo and MCF-10A/ERα36 cells. Histogram depicts the wound healing when measured after a 6-hour culture (left panel). A representative picture of the migrating MCF-10A/ERα36 cells is presented in the right panel. Each bar represents mean ± S.E.M. N = 5. *: *P* <0.05. B-C. MCF-10A/Zeo and MCF-10A/ERα36 were cultured for 24 hours. B. CDH1 and CDH2 gene expression was measured by RT-PCR analysis. The housekeeping gene RPLPO was used as the reference gene. Each bar represents mean ± S.E.M. N = 3. *: *P* <0.05. **: *P* <0.01. C. Expression of cell-cell adhesion proteins was studied by immunofluorescence with specific antibodies: anti-β-catenin, anti-E-cadherin and anti-N-cadherin (AlexaFluor 555). Average fluorescent signal intensities are quantified from 5 cells from 5 fields per condition. Each bar represents mean ± S.E.M. N = 3. *: *P* <0.05, ** *P* <0.01.

Using the scratch-wound assay, we observed an accelerated closure of the wound created in a confluent monolayer culture of MCF-10A/ERα36 compared to MCF-10A/Zeo cells (Fig 4A). Therefore, expression of epithelial-mesenchymal transition markers was analyzed by RT-PCR. CDH1 expression was reduced by 70% whereas CDH2 was induced 50% by ERα36 overexpression (Fig 4B).

Expression and localization of proteins known to be involved in cell-cell junctions were also examined: ERα36 overexpression triggered the decrease of E-cadherin expression (15%) and beta-catenin (30%) membrane immunofluorescence staining toward a dispersed cytoplasmic localization whereas N-cadherin staining was significantly augmented by 36% (Fig 4C).

### Consequences of ERα36 expression in the mouse mammary gland

**Fig 5:**
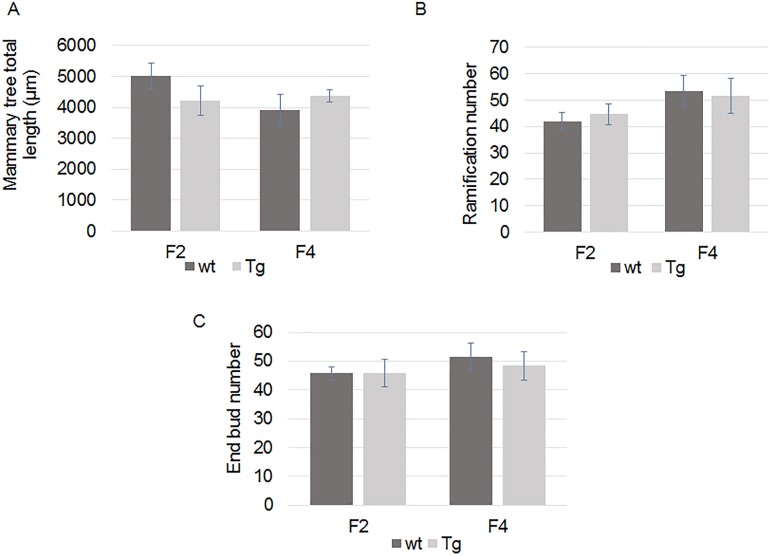
Whole mount mammary tree parameters analyses in wt and Tg mice at weaning (PND21). F2 or F4 mammary glands from wt or Tg mice where mounted. Computational analysis of mammary tree total extension (A), number of branching (B) or end buds (C) was performed using a dedicated software. No significant difference was observed between wt and Tg mice. F2wt: N = 4, F2 Tg: N = 6, F4wt: N = 6, F4Tg N = 5.

We hypothesized that ERα36 overexpression could be sufficient to promote mammary epithelium alteration in vivo. Since ERα36 sequence is absent in mouse, we produced ERα36 transgenic mice in which ERα36 is expressed under the control of MMTV (Mouse Mammary Tumor Virus) promoter, specifically in the mammary gland [29]. Mammary tree structure of wild-type (wt) and transgenic (Tg) mice was analyzed with a dedicated software designed to skeletonize mammary gland RGB images and compute mammary tree extension (Fig 5A), number of branching (Fig 5B) and end buds (Fig 5C). This study was completed by histological analyses of mammary gland slices and western blot analyses of protein expression.

**Fig 6:**
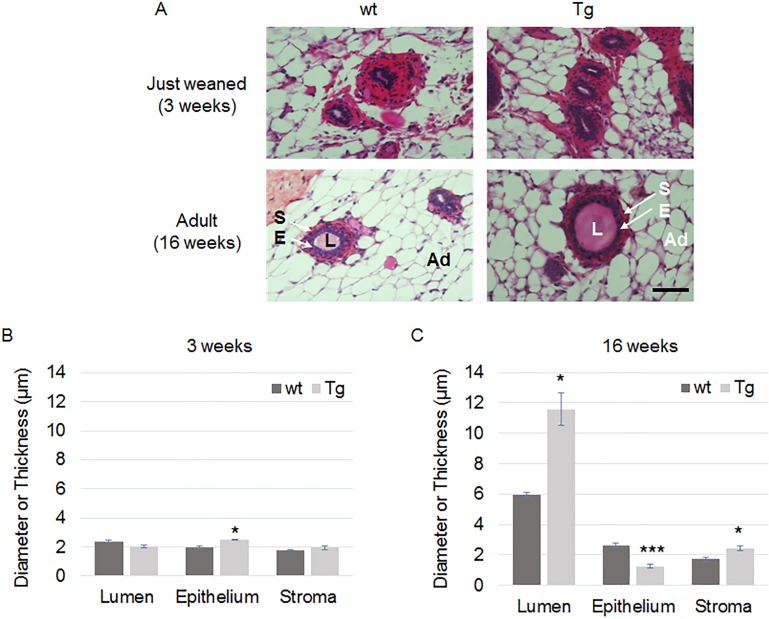
Histological analysis of F4 wt and Tg mice at weaning and adulthood. F_4_ mammary gland slices from 3 week old (weaning) or 16 week old (adult) virgin female wt or Tg mice were stained with hematoxylin/eosin (A). S, stroma; E, epithelium; L, lumen; Ad, adipocyte. The epithelium thickness, the stromal thickness and lumen diameter of mammary ducts were measured in wt or Tg mice at weaning (B) and adulthood (C). Scale bar, 15 μm. Each bar represents mean ± S.E.M. N>5 per group. *: *P* <0.05, ***: *P* <0.001.

First, we addressed the effects of an ERα36 expression on fetal and neonatal mammary gland development by exploring the differences between wt and Tg mammary glands harvested on F2 or F4 mice at weaning and adulthood (Fig 6A). Neither mammary tree parameters nor measurement of epithelium or stroma thickness or lumen diameter varied at weaning (PND21) between wt and Tg animals (Fig 6B).

Conversely, at adulthood (16 week old), lumen diameter (*P* = 0.0036) and stroma thickness (*P* = 0.0029) were significantly augmented whereas epithelium thickness (*P* = 9x10^-7^) was reduced in F2, F3 and F4 Tg female mice compared to wt ones (Fig 6C). This phenotype correlated with loss of E-cadherin expression in mammary glands from transgenic mice (S6 Fig).

These results suggest that ERα36 expression promotes mammary gland alteration after the puberty when potential estrogen receptor ligands such as steroid hormones are endogenously produced or reach the targeted mammary gland.

## Discussion

The data presented herein depict an integrated picture of normal mammary epithelial cells overexpressing ERα36. In the human MCF-10A cell line, ERα36 modulates the expression of genes that control proliferation, survival and migration. The pathways inferred from these data by computational modeling indicate that the regulation of those ERα36 target genes should involve preferentially the JAK2/STAT3 pathway as previously described in breast cancer cells in response to estrogens or anti-estrogens [13, 37]. These data are in line with the prior assumption of Björnström and Sjöberg [38] and Marino [39] who proposed that estrogenic signaling takes place through multiple pathways. Indeed, Zhang and Wang [13] and Fox [40] reported an ERα36 dependent control of proliferation and resistance to apoptosis through SRC/EGFR/STAT3/5 in breast cancer cells. Since ERα36 has been shown to collaborate with other estrogen or growth factor receptors in numerous cancer cells, our results also raise the possibility that an excess of ERα36 protein could be a dominant positive activator of either the G protein-coupled estrogen receptor GPER, or the EGFR downstream signaling. Indeed, GPER mRNA expression was stimulated by 2.09 fold in MCF-10A cells overexpressing ERα36. Moreover, ERα36 was shown to interact physically with GPER [41, 42] and/or to collaborate with GPER to trigger downstream signaling in female reproductive tract, seminoma or breast cancer cells [5, 16, 17, 43, 44]. JAK2/STAT3 signaling was also reported to be involved in GPER signaling in the hypothalamus and SKBR-3 breast cancer cells [45, 46]. EGFR mRNA expression was not affected by ERα36 overexpression in normal epithelial cells (data not shown) whereas a positive cross-activation of both gene expression has been reported in seminoma and breast cancer cells [16, 41, 42]. Since EGF is present in cell culture medium, ERα36 protein could enhance a basal level of EGFR signaling through PI3K/AKT and STAT3/5. In vivo, a combination of EGF and progesterone treatment of ERαKO mice was shown to partially sustain mammary tree elongation and to modulate cytokine-cytokine receptor interaction (namely IL6/JAK2/STAT3 signaling), adhesion, TGFβ signaling and apoptosis, four functions identified in the functional analysis of DEGs [47].

The ERα36 dependent stimulation of STAT3 expression and nuclear localization is of particular interest since constitutively activated STAT3 has been demonstrated to directly contribute to oncogenesis in various cancers by enhancing cell proliferation and migration but preventing cell apoptosis [48]. Indeed, STAT3 is supposed to be a promising therapeutic target of novel anticancer drugs like methyl ester derivative of synthetic triterpenoid which have been tested in a relevant model of ER negative breast cancers [49]. Together with NF-kB, which expression is also increased in ERα36 overexpressing cells, STAT3 has been shown to play a pivotal role in the epigenetic switch linking inflammation to mammary gland tumorigenesis (i) in a model of MCF-10A cells containing and ER-src fusion exposed to tamoxifen as well as (ii) in the pre-tumorigenic state of mammary epithelium from mice constitutively overexpressing ERBB2 [50–52].

In this study, an in vivo mammary specific ERα36 overexpression was performed through the production of a unique MMTV-ERα36 transgenic mouse strain. At weaning, no difference could be observed at the microscopic level in the mammary gland between transgenic and wt mice. However, in ERα36 overexpressing adult virgin females, the mammary gland displayed a significative dilation of the ducts as well as stromal thickening and epithelium thinning and leakage. A similar but limited dilation was also reported by Bocchinfuso [53] after exogenous estradiol treatment of wild-type mice. These results suggest that the endogenous estrogens produced from puberty in ERα36 transgenic mice could signal trough the ERα36 protein and mimic the overstimulated mammary estrogenic response observed in estradiol exposed wt mice. Since ERα36 is able to down regulate ERα66 expression or to behave as a dominant negative of ERα66 activity [54], the abnormal epithelium and stromal phenotype could result from an alteration of the correct estrogen signaling pathway. In conclusion, molecular, cellular and tissue phenotype of ERα36 overexpressing models evoke a constitutive estrogenic stimulation. Noteworthy, epithelium thinning and leakage are in line with lower proliferation rate and enhanced migration observed in vitro, respectively.

To date, commercially available antibodies gave poor results in our hands for the in situ detection of ERα36 receptor in normal or cancerous mammary tissues. However, a basal expression of the ERα36 mRNA (and sometimes of the protein) has been detected in many organs [18]. Since a high level of active ERα36 is known to be one of the main actors of breast cancer progression [18], it would be noteworthy to investigate if ERα36 function in cancer requires either an expression or/and an agonist concentration threshold. Indeed, our data from normal mammary epithelial cells suggest that, in the absence of ligand, ERα36 overexpression is sufficient to trigger cell migration and partial resistance to apoptosis, two hallmarks of cancer cells. In the presence of a physiological adult level of estradiol, ERα36 also mediates mammary epithelium disorganization in vivo, indicating a circuitous estrogen signaling in the presence of a high ERα36 receptor level.

We previously demonstrated that estrogen mimicking compounds such as long chain alkylphenols stimulate GPER/ERα36 signaling pathways in human seminoma cells [17]. Many papers, namely from Ana M Soto’s laboratory also demonstrate that fetal or neonatal exposure to BPA or DES alter the estrogen signaling in the developing mouse or rat mammary gland of rodents and predispose animals to develop breast neoplasia later in adulthood [55]. Therefore, the unique model of MMTV-ERα36 transgenic mice described in this study could serve to screen endogenous or xenobiotic molecules, acting as ERα36 agonists or antagonists and to decipher their mechanisms of action. How and when ERα36 expression is initiated and maintained in mammary epithelial cells remain to be investigated.

## Supporting information

S1 Fig Production of ERα36 transgenic mice. A. Structure of ERα36 transgene. MMTV promoter was cloned from the pGL4.36[luc2P/MMTV/Hygro] reporter vector (Promega, France) and placed upstream ERα36 complete cDNA sequence and a poly(A) stretch. Construction was then microinjected into B6SJLF2 fertilized eggs.
B. ERα36 protein expression in transgenic adult mouse. Western blot analysis of ERα36 protein expression in adult mammary gland (4 month old females). wt: wild-type; Tg: transgenic. Tubulin protein expression is used as loading control.
C. Sex ratio and transgene transmission rate in ERα36 transgenic strain. The litters from 27 wild-type females mated with ERα36 KI hemizygote males were analyzed for sex ratio and transgene transmission rate. Among 171 animals, 47.4% were males and 52.6% were females. A non-significant (*P* = 0.16) lack of transgene transmission was observed, especially in females (40% measured *versus* 50% expected transgene transmission rate using Chi2 test).
(TIF)

S2 Fig Mammary tree parameter analyses. Mammary tree whole mounts images (A) from PND21 mice were skeletonized with a dedicated software (B). Total extension (white line), number of duct branching (green spots) and sprouts (red spots) were computed.
(TIF)

S3 Fig Characterization of MCF-10A/ERα36 cell line. A. Real-time PCR analysis of ERα36 expression in MCF-10A/ERα36 and MCF-10A/Zeo cells. The ERα36 mRNA expression level slightly detected by real-time PCR in MCF-10A/Zeo cells was set to 1. Several clones of MCF-10A/ERα36 cells were tested for ERα36 expression which varied from 2- to 42-fold the one of MCF-10A/Zeo cells. For further analyses, we selected the 36–4 clone in which ERα36 expression was augmented by 13-fold compared to MCF-10A/Zeo cells. This overexpression level was in the range of the difference observed between MCF-10A cells and “naturally ERα36 expressing” MDA-MB-231 breast cancer cells (Zou et al; 2009 [54]).
B. Western-blot analysis of ERα36 in MCF-10A/Zeo and MCF-10A/ERα36. ERα36 protein is not detectable by western blotting in MCF10A/Zeo cells. However, ERα36 expression is revealed by an anti-ERalpha antibody (G20) in MCF-10A/ERα36 cells (clone 36–4 selected from panel A).
C. Immunoflorescence analysis of ERα36 expression and localization in MCF-10A/ERα36 and MCF-10A/Zeo cells. Merge images show nuclei stained blue with Hoechst and ERα36 stained red by anti-ERα36 rabbit polyclonal primary antibody and anti-rabbit-Alexafluor 555 secondary antibody (clone 36–4 selected from panel A). Scale bar = 50μm.
(TIF)

S4 Fig Overall strategy of microarray data bioinformatic analysis. A. Differentially expressed genes (DEGs) from MCF-10A/ERα36 and MCF-10A/Zeo cells were submitted to MSigDB ^®^ online tool to performed functional analyses. This led to the identification of the functions and signaling pathways mostly affected by ERα36 overexpression. Ingenuity pathway analysis (IPA ^®^) determined the DEG upstream regulators and a dedicated Matlab^®^ software was designed to identify intermediate regulators acting downstream ERα36 and upstream DEGs.
B. Example of one hierarchized gene network built by iterative computation of resulting adjacency matrices with a dedicated software from Matlab^®^.
(TIF)

S5 Fig ERα36 overexpression modulates MAPK/Erk1/2, NFκB and JAK2/STAT3 signaling pathways in MCF-10A cells. A. Representative western blot analysis of Phospho-Erk1/2 (P-Erk), total Erk1/2 (t-Erk) PTEN, NFκB and STAT3 expression in MCF-10A/Zeo and MCF-10A/ERα36 cells. β-Actin was used as a loading control.
B. Localization of NFκB and STAT3 was studied by immunofluorescence with specific antibodies: anti-NFκB p65, anti-STAT3 (red, AlexaFluor 555). Hoechst was used to stain the nuclei (blue). A nuclear translocation of NF-κB and STAT3 was observed in MCF10A/ERα36 cells compared to MCF10A/Zeo cells. Scale bar = 50μm.
(TIF)

S6 Fig ERα36 overexpression triggers loss of E-cadherin expression in adult transgenic mice. Representative western blot analysis of E-cadherin expression in wt and Tg mammary glands. α-tubulin was used as a loading control.
(TIF)
