# Supplementary figures and images for: Mammary epithelial cell phenotype disruption in vitro and in vivo through ERalpha36 overexpression
Source: PLoS One. 2017 Mar 16;12(3):e0173931. doi: 10.1371/journal.pone.0173931 (PMC5354400; doi:10.1371/journal.pone.0173931)

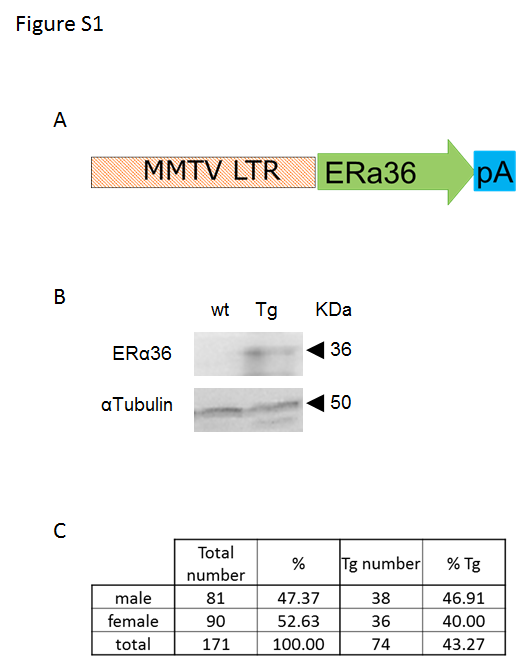

Supplement: S1 Fig — A. Structure of ERα36 transgene. MMTV promoter was cloned from the pGL4.36[luc2P/MMTV/Hygro] reporter vector (Promega, France) and placed upstream ERα36 complete cDNA sequence and a poly(A) stretch. Construction was then microinjected into B6SJLF2 fertilized eggs. B. ERα36 protein expression in transgenic adult mouse. Western blot analysis of ERα36 protein expression in adult mammary gland (4 month old females). wt: wild-type; Tg: transgenic. Tubulin protein expression is used as loading control. C. Sex ratio and transgene transmission rate in ERα36 transgenic strain. The litters from 27 wild-type females mated with ERα36 KI hemizygote males were analyzed for sex ratio and transgene transmission rate. Among 171 animals, 47.4% were males and 52.6% were females. A non-significant (P = 0.16) lack of transgene transmission was observed, especially in females (40% measured versus 50% expected transgene transmission rate using Chi2 test). (TIF) [file pone.0173931.s001.tif]

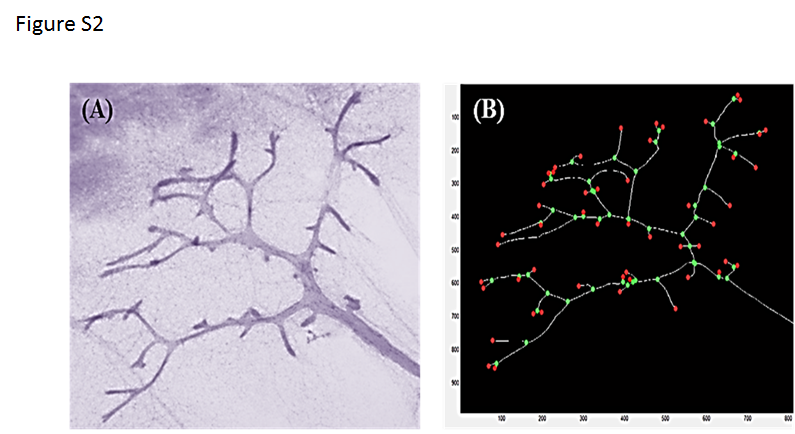

Supplement: S2 Fig — Mammary tree whole mounts images (A) from PND21 mice were skeletonized with a dedicated software (B). Total extension (white line), number of duct branching (green spots) and sprouts (red spots) were computed. (TIF) [file pone.0173931.s002.tif]

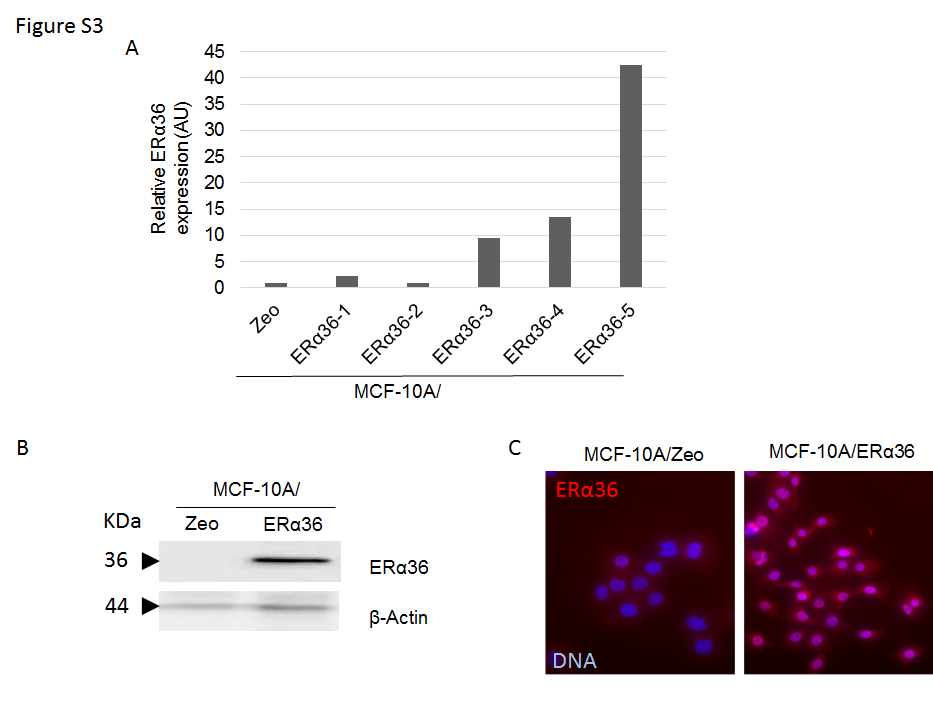

Supplement: S3 Fig — A. Real-time PCR analysis of ERα36 expression in MCF-10A/ERα36 and MCF-10A/Zeo cells. The ERα36 mRNA expression level slightly detected by real-time PCR in MCF-10A/Zeo cells was set to 1. Several clones of MCF-10A/ERα36 cells were tested for ERα36 expression which varied from 2- to 42-fold the one of MCF-10A/Zeo cells. For further analyses, we selected the 36–4 clone in which ERα36 expression was augmented by 13-fold compared to MCF-10A/Zeo cells. This overexpression level was in the range of the difference observed between MCF-10A cells and “naturally ERα36 expressing” MDA-MB-231 breast cancer cells (Zou et al; 2009 [54]). B. Western-blot analysis of ERα36 in MCF-10A/Zeo and MCF-10A/ERα36. ERα36 protein is not detectable by western blotting in MCF10A/Zeo cells. However, ERα36 expression is revealed by an anti-ERalpha antibody (G20) in MCF-10A/ERα36 cells (clone 36–4 selected from panel A). C. Immunoflorescence analysis of ERα36 expression and localization in MCF-10A/ERα36 and MCF-10A/Zeo cells. Merge images show nuclei stained blue with Hoechst and ERα36 stained red by anti-ERα36 rabbit polyclonal primary antibody and anti-rabbit-Alexafluor 555 secondary antibody (clone 36–4 selected from panel A). Scale bar = 50μm. (TIF) [file pone.0173931.s003.tif]

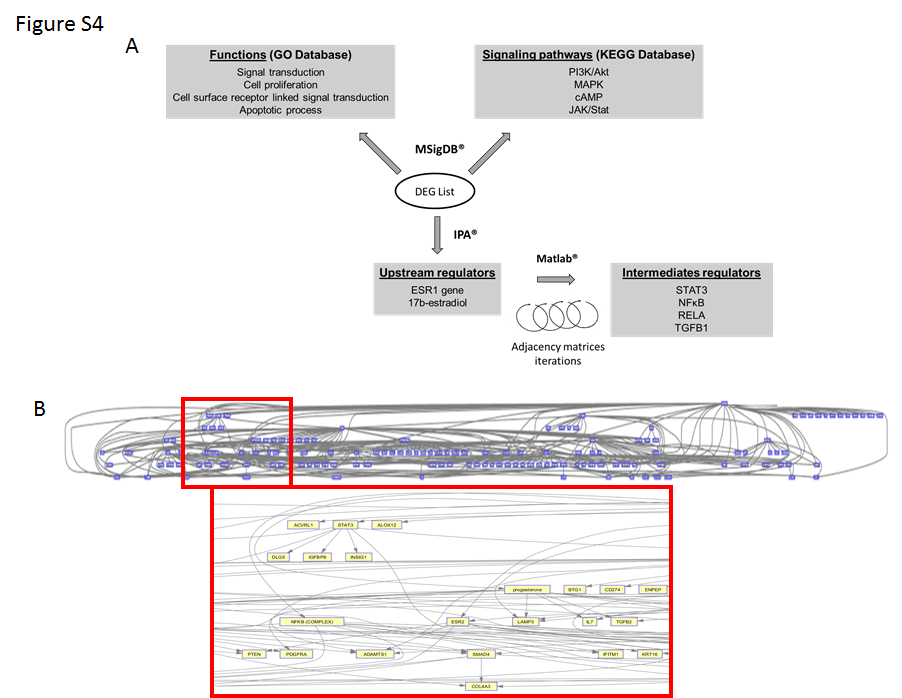

Supplement: S4 Fig — A. Differentially expressed genes (DEGs) from MCF-10A/ERα36 and MCF-10A/Zeo cells were submitted to MSigDB ® online tool to performed functional analyses. This led to the identification of the functions and signaling pathways mostly affected by ERα36 overexpression. Ingenuity pathway analysis (IPA ®) determined the DEG upstream regulators and a dedicated Matlab® software was designed to identify intermediate regulators acting downstream ERα36 and upstream DEGs. B. Example of one hierarchized gene network built by iterative computation of resulting adjacency matrices with a dedicated software from Matlab®. (TIF) [file pone.0173931.s004.tif]

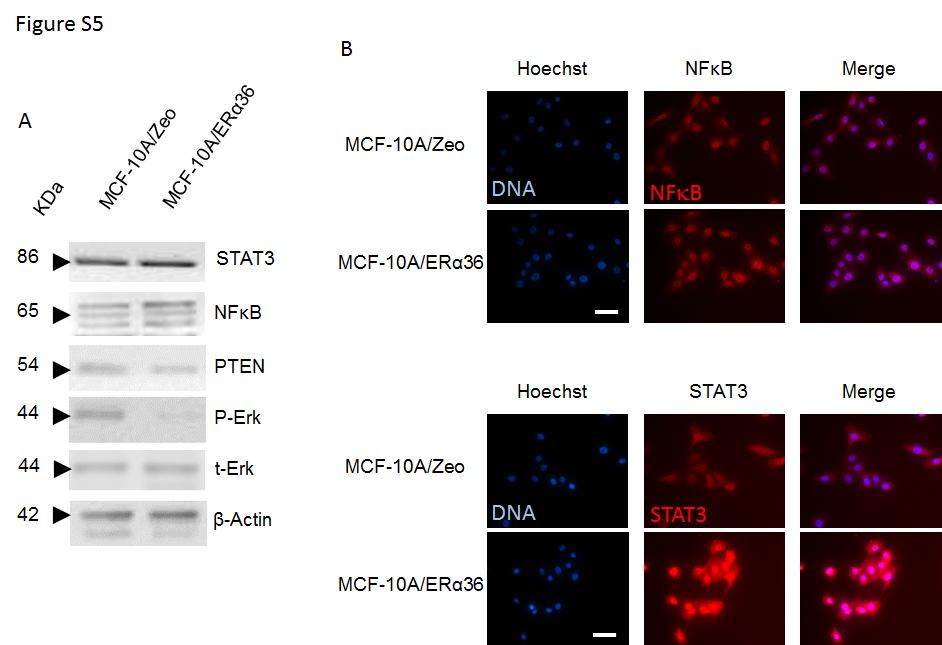

Supplement: S5 Fig — A. Representative western blot analysis of Phospho-Erk1/2 (P-Erk), total Erk1/2 (t-Erk) PTEN, NFκB and STAT3 expression in MCF-10A/Zeo and MCF-10A/ERα36 cells. β-Actin was used as a loading control. B. Localization of NFκB and STAT3 was studied by immunofluorescence with specific antibodies: anti-NFκB p65, anti-STAT3 (red, AlexaFluor 555). Hoechst was used to stain the nuclei (blue). A nuclear translocation of NF-κB and STAT3 was observed in MCF10A/ERα36 cells compared to MCF10A/Zeo cells. Scale bar = 50μm. (TIF) [file pone.0173931.s005.tif]

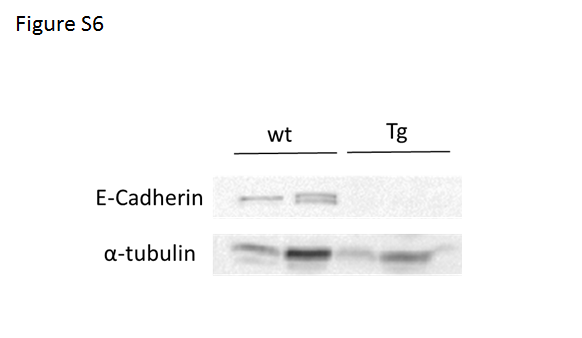

Supplement: S6 Fig — Representative western blot analysis of E-cadherin expression in wt and Tg mammary glands. α-tubulin was used as a loading control. (TIF) [file pone.0173931.s006.tif]
